# Two-Item Fall Screening Tool Identifies Older Adults at Increased Risk of Falling after Emergency Department Visit

**DOI:** 10.5811/westjem.2020.5.46991

**Published:** 2020-08-20

**Authors:** Christopher J. Solie, Morgan B. Swanson, Kari Harland, Christopher Blum, Kevin Kin, Nicholas Mohr

**Affiliations:** University of Iowa Hospitals and Clinics, Department of Emergency Medicine, Iowa City, Iowa

## Abstract

**Introduction:**

Few emergency department (ED)-specific fall-risk screening tools exist. The goals of this study were to externally validate Tiedemann et al’s two-item, ED-specific fall screening tool and test handgrip strength to determine their ability to predict future falls. We hypothesized that both the two-item fall screening and handgrip strength would identify older adults at increased risk of falling.

**Methods:**

A convenience sample of patients ages 65 and older presenting to a single-center academic ED were enrolled. Patients were asked screening questions and had their handgrip strength measured during their ED visit. Patients were given one point if they answered “yes” to “Are you taking six or more medications?” and two points for answering “yes” to “Have you had two or more falls in the past year?” to give a cumulative score from 0 to 3. Participants had monthly follow- ups, via postcard questionnaires, for six months after their ED visit. We performed sensitivity and specificity analyses, and used likelihood ratios and frequencies to assess the relationship between risk factors and falls, fall-related injury, and death.

**Results:**

In this study, 247 participants were enrolled with 143 participants completing follow-up (58%). During the six-month follow-up period, 34% of participants had at least one fall and 30 patients died (12.1%). Fall rates for individual Tiedemann scores were 14.3%, 33.3%, 60.0% and 72.2% for scores of 0,1, 2 and 3, respectively. Low handgrip strength was associated with a higher proportion of falls (46.3%), but had poor sensitivity (52.1%).

**Conclusion:**

Handgrip strength was not sensitive in screening older adults for future falls. The Tiedemann rule differentiated older adults who were at high risk for future falls from low risk individuals, and can be considered by EDs wanting to screen older adults for future fall risk.

## INTRODUCTION

### Background

Falls are the leading cause of traumatic mortality in older adults.^1^,^2^ Nearly three million older adults are seen annually in United States emergency departments (ED) for falls.^3^ Each year 33% of community-dwelling adults over the age of 65 experience a standing level fall.^4^ Of patients admitted to the hospital with a ground level fall, 44% are readmitted and 33% die within one year.^5^–^7^ Those who present to the ED with fall-related injuries and are discharged have higher rates of future falls, functional decline, and additional ED visit within three months than other older adults.^8^,^9^

### Goals

A recent review looked at thresholds both for testing individuals for fall risk and treating with fall prevention therapies.^10^ The authors concluded that individuals having a 27% risk of falling in the next six months should receive fall prevention interventions. Our goal was to evaluate two tools, which can feasibly be performed in an ED, to determine whether they can appropriately risk stratify patients’ fall risk above the treatment threshold of 27% with good sensitivity. The primary objective of this study was to measure the ability of the two-item fall screening tool previously devised and internally validated by Tiedemann et al and handgrip strength to predict future six-month fall risk in adults 65 years and older presenting to the ED.^11^ Secondary objectives were to assess both tools’ ability to predict fall-related injury and/or death within six months.

## METHODS

### Study Design

This study was a single-center, prospective observational cohort study of ED patients. It was approved by the local institutional review board, and written informed consent was obtained from all participants.

### Study Setting and Population

All study enrollments were performed at a single Midwestern academic ED with an annual patient volume of about 65,000 patients annually. Patients were eligible for participation in the study if they were 65 years of age or older and were treated in the ED between 9 am and 11:59 pm on weekdays and 2 pm and 10 pm on weekends. We excluded from the study patients currently living in a nursing home, prisoners, patients with limited English-language skills, and those without the capacity to provide informed consent. If a participant moved to a nursing home after the time of consent, but during the study follow-up period, they remained in the study. No compensation was provided to study participants. The study is reported in accordance with the Strengthening the Reporting of Observational Studies in Epidemiology (STROBE) Statement: guidelines for reporting observational studies.^12^

### Study Protocol

Qualifying patients presenting to the ED were consented for the study by a trained research assistant during their ED visit from March 2017–June 2017. Participants provided basic demographic information, completed self-administered fall screening surveys, and provided handgrip strength measurements. Following ED discharge, patients were mailed six follow-up postcards at consecutive monthly intervals from one month to six months after their ED visit. Participants who did not respond to the monthly postcard were then contacted by email or phone (based on patient preference).

**Population Health Research Capsule**

What do we already know about this issue?

Few ED-specific fall-risk screening tools exist, making it difficult to identify high-risk individuals who would benefit from fall prevention therapies.

What was the research question?

Would low handgrip strength and a 2-item fall screening tool be able to accurately predict future 6-month fall risk in older adults presenting to the ED?

What was the major finding of the study?

The two-item screening tool identified those at high risk of future falls with good sensitivity.

How does this improve population health?

Future falls for older adults identified by a screening tool during the ED visit could be decreased by referral to fall prevention therapies.

### Measurements

At the time of the ED visit, screening questions from Tiedemann et al were used to assess geriatric fall risk.^11^ Using Tiedemann’s rule, as done in the previous validation study, having two or more falls within the prior year was worth two points and taking six or more medications was worth one point (Table 1). Handgrip strength was measured using a handgrip dynamometer (Constant 200 lbs. Digital Hand Dynamometer Grip Strength Measurement Meter, Camry Electronic Ltd, Guangdong, China) on both hands in kilogram-force (kgf), and handedness was also reported by the patient.

### Key Outcome Measures

The primary outcome was any fall within six months. Secondary outcomes included fall-related injury and/or death during the six-month follow-up period. Falls and fall-related injuries were ascertained from participant self-report from monthly follow-up (postcard, email, or phone call). In the monthly follow-up, participants were asked, “Have you fallen within the past month?” for falls and, if they had fallen, “Were you injured?” for fall-related injuries. Death was determined from a combination of family or friend report during monthly follow-up, and participants lost to follow up were screened in the state death registry to identify cases where loss to follow-up was because of death.

### Data Analysis

Demographic information was reported across fall status using descriptive statistics including the chi-square tests and Mann-Whitney U tests, as appropriate. Test charactersitics (sensitivity, specificity, likelihood ratios, and diagnostic odds ratios) for the Tiedemann rule were calculated for each Tiedemann score (0,1,2, and 3). We generated a receiver operating characteristic (ROC) curve to evaluate the predictive value of the Tiedemann score for six-month fall within the study population. To generate 95% confidence intervals (CI) for the area under the curve (AUC) estimates for the ROC curve, we randomly generated 1000 study samples (with replacement of observations) from the study data by bootstrapping. This provided an estimate of the variation in the AUC point estimate. Similar analyses were completed for the secondary outcomes of fall-related injury and composite fall or death.

Handgrip strength was reported as mean and 95% CI by dominant hand and compared between the those who fell and those who did not. We used stratification by gender, as there were observed differences in the distributions of handgrip strength by gender. A ROC curve was generated using similar techniques to those described above, including internal validation of the AUC with bootstrapping, to evaluate the predictive value of handgrip strength for six-month fall. A threshold value for low handgrip strength was selected by identifying the handgrip value for each gender that maximized specificity and sensitivity. Low handgrip strength was defined as less than 16 kg for females and less than 25 kg for males. Using the dichotomized measure, we calculated sensitivity, specificity, likelihood ratios, and diagnostic odds ratios to determine the prognostic utility of low handgrip strength for six-month fall. Similar analysis was completed for both secondary outcomes.

To account for high loss to follow-up, we conducted a post hoc survival analysis using interval censoring assessed for differences in fall-free survival by Tiedemann screening status. A composite outcome of death or fall was used to define the outcome. Participants were right-censored at the end of the six-month follow-up period or after loss to follow-up (i.e, a missed monthly survey). Survival function estimates curves were constructed to visualize fall-free survival, median survival time was computed for each group, and differences in fall-free survival rates tested for significance with the log-rank test, incorporating interval censoring.

### Sample Size Calculation

Assuming an alpha of 0.05, power of 0.80, prevalence of a fall within six months of 17%^13^ and test sensitivity of 93% and specificity of 61% for a fall within six months,^13^ 147 participants were needed for analysis. Assuming 40% lost to follow-up based upon a previous ED study with similar follow-up methods,^13^ 245 participants were needed to have a final analysis sample of 147 participants.

### Sensitivity Analysis

We performed a sensitivity analysis incorporating all participants, including those lost to follow-up at six months,. Participants lost to follow-up were assumed to not have fallen (outcome = 0) for the sensitivity analysis.

### Missing Data

For the primary analysis describing the test characteristics of the fall screening tools, we used complete case analysis. The complete case analysis population included participants who responded to the monthly follow-up surveys at month six; deaths were included in the loss-to-follow-up population for this analysis. For the outcome of death or fall, participants who died during the study period were also included. For the survival analysis, we included participants from study enrollment until the first event (fall or death) or until right-censoring occurred (ie, end of six-month study period or lost to follow-up). Data from participants who partially completed follow-up were compared to those completing all of follow-up to examine how those lost to follow-up might differ from those who completed six-month follow-up. We conducted all data analysis in SAS version 9.4 (SAS Institute, Cary, NC).

## RESULTS

### Decription of Study Population

**Figure 1:**
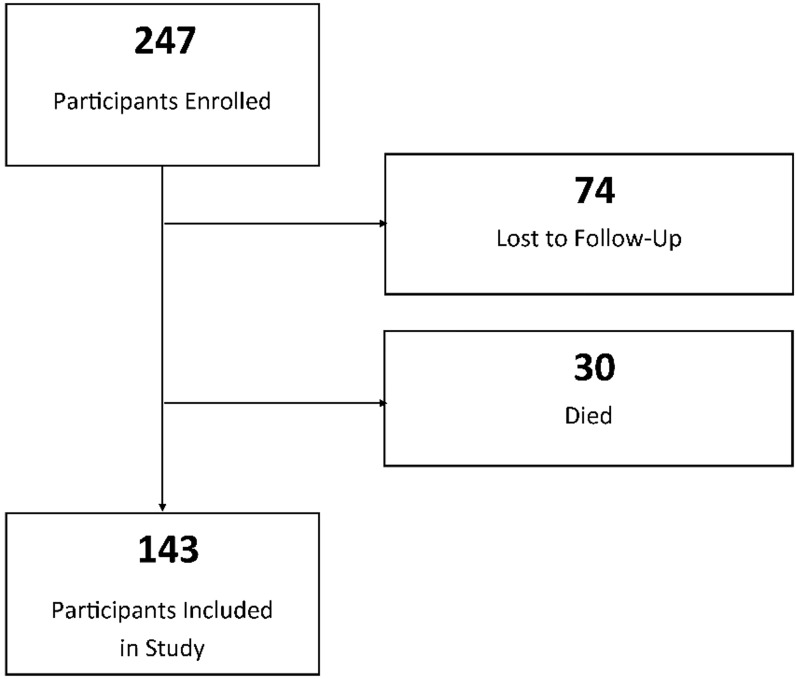
Flowchart of enrollment in study of tool to predict future falls in older adults. *For survival analyses and composite outcome, 30 participants who died are included in analysis (n = 173)

A total of 247 patient were enrolled, with 74 patients (30%) lost to complete six months of follow-up (Figure 1). Thirty participants died during the study. There were 194 participants who completed at least part of the follow up. The final six-month fall analysis included 143 participants.

Demographics of our study participants can be found in Table 2. The median age was 74 years. Men made up 47% of the study population. There were no major differences in age, gender, dominant or non-dominant handgrip strength or fall-related visits in those lost to follow-up (Supplemental Table S1). During initial ED evaluation 23 (16%) patients reported two or more falls in the past year and 96 (67%) reported taking six or more medications (Table 3). Low handgrip strength was found in 54 (38%) patients.

### Primary Outcome: Falls

There were 48 (34%) participants who had a fall within six months of their ED presentation with a total of 107 reported falls. A Tiedemann score of 0 had a 14.3% fall rate. Tiedemann scores of 1,2 and 3 had fall rates of 33.3%, 60.0% and 72.2%, respectively. A score of 1 or greater was sensitive (87.5%, 95% CI, 78.1 – 96.9 ) in identifying those who fell. Higher score thresholds were more specific for future fall risk; however, they were poorly sensitive (Table 4).

**Figure 2:**
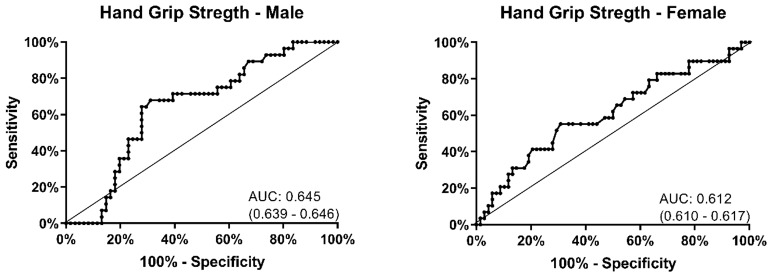
Receiver operating characteristic curve for handgrip strength. *AUC*, area under the curve.

There was no difference in handgrip strength between the group that fell and the group that did not (Table 2). Participants with low grip strength did have a high fall rate (46%), but this was poorly sensitive (52%, 95% CI, 38.0–66.2) and specific (69%, 95% CI, 60.2–78.7) (Table 4). The receiver operating area under the curve for handgrip strength was 0.645 (95% CI, 0.639 – 0.646) in men and 0.612 (95% CI, 0.610 – 0.617) in women (Figure 2). There was no threshold for hand grip strength found to identify those at greatest risk of falling.

### Secondary Outcomes: Death and Injury

**Figure 3:**
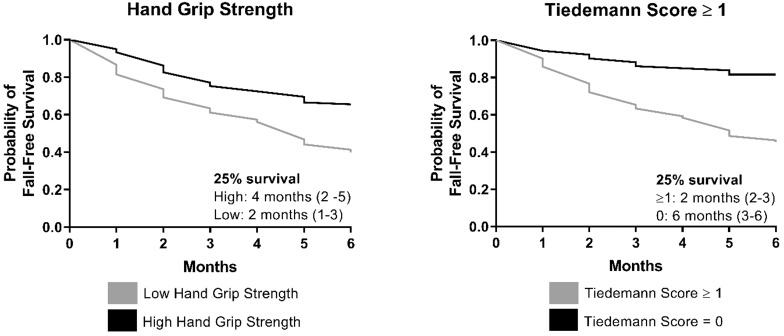
Interval-censored Kaplan-Meier plots by screening test results.

Of the participants who reported a fall, 54% had a fall-related injury. The Tiedemann rule was able to distinguish low risk from high risk partipants for fall-related injuries (Table 4). The percentage of participants who fell and had a fall-related injury was nearly identical in those with a negative Tiedemann score vs those with a score of 1 or greater (50% vs 54%). There were 30 (12.1%) participants who died during their six-month follow-up period. The Tiedemann rule also performed well in risk stratifying those at risk of fall or death. Patients with a Tiedemann score of 0 had a significantly greater probability of a fall-free survival at six months (Figure 3). Patients with scores of 1 or greater had a significantly lower probability of a fall-free survival.

Fall-related injuries were higher in the group that had low handgrip strength (27.8% vs 18.2%). The low handgrip strength group had a higher rate of fall and/or death at six months (40.0% vs 24.2%) and a lower rate of fall-free survival (Figure 3).

## DISCUSSION

Falls are a major problem for older adults and our health system.^1^–^9^ Identifying older adults at risk of future falls is important as interventions have proven to decrease fall risk. Many of these interventions involve referral and home-based assessment, which can be coordinated through the ED. In the US, fall-prevention programs are often offered by senior community centers, YMCAs and physical therapists. Many programs focus on balance, strength training, and environmental changes. The Prevention of Falls in the Elderly Trial proved that ED treatments can prevent future falls. This study randomly assigned high risk older adults to a fall-intervention program, and participation in the program decreased future falls from 52% to 32%.^14^

Recognizing the importance of identifying those at increased risk for future falls, the Society of Academic Emergency Medicine Geriatric Emergency Medicine Residency Core Competencies and the “Geriatric Emergency Department Guidelines” recommend that geriatric patients be screened for fall risk, although there is currently no specific screening tool recommended.^15^–^17^ While many fall-screening tools exist, few have been evaluated in an ED setting. A review of ED-specific fall-screening tools identified only two studies that derived ED-specific fall-screening tools using individual risk factors with six-month falls as the primary outcome.^10^ Extensive fall risk evaluations are not feasible in EDs, but brief screening programs hold promise. Our study found that simply asking two questions can distinguish those at high risk of falling from those at lower risk. This is the first ED-specific fall-risk screening tool to be externally validated.

In our study, the Tiedemann rule was able to distinguish those at high risk of falling from those at low risk. The tool also performed well in identifying those at increased risk of fall-related injury and fall or death. Asking two questions enabled care providers to distinguish those who would benefit from fall prevention interventions with good sensitivity (87.5%, 95% CI, 78.1 – 96.9). Older adults with a Tiedemann score of 0 had such a low fall rate (14.3%) that sending these patients to fall prevention therapies would likely have been of little benefit. A score of 1 or higher had a combined fall rate of 41.6%. Using the treatment threshold of 27% previously described by Carpenter et al, we recommend EDs using the Tiedemann two-question screening tool refer those with a score of 1 or greater to fall-prevention interventions.

Frailty is the state of vulnerability due to poor resolution of homeostasis as a response to a stressor event and has been found to put older adults at greater risk for falls.^18^–^20^ Of the many proposed ways to measure frailty, one of the simplest is handgrip strength, which has been shown to be a single marker for frailty, more than chronological age itself.^21^ Handgrip strength is measured with a hand dynamometer, which is a non-invasive, inexpensive device (approximately $25) that can perform the measurement in seconds. As decreased handgrip strength has been used to identify frailty and frailty has been associated with increased risk of falls, we predicted that decreased handgrip strength would be able to predict increased risk of future falls. In our study, low handgrip strength was associated with an increased risk of fall, fall-related injury and fall or death at six months, but did not perform well as a fall-risk screening tool as it failed to identify almost half of those who fell.

While checking handgrip strength in the ED may not be useful as a fall-risk screening tool, there may be other benefits from checking handgrip strength as it did identify a more frail subgroup of older adults given increased rates of future injury and death. This could help in adding objective data for supporting an individual’s need for nursing home placement. Future studies are needed to evaluate its utility in the ED.

## LIMITATIONS

This study has several limitations. The biggest limitation was our loss to follow-up. Although our loss to follow-up was high, it was lower than our predicted loss to follow-up of 40%; thus, we met our goal sample size. Those lost to follow-up had lower handgrip strength and had a higher incidence of falls contributing to their index visit (Supplemental Table S1). We had anticipated a lower fall rate, as reported in another US-based ED study,^13^ but our fall rate was similar to that found in Tiedemann et al’s study.

Our study was performed at a single academic center that primarily serves a White, non-urban population. These findings may not reflect EDs that serve other demographic groups as our population may have different fall hazards than older adults in more urban locations. However, the consistency between our results and the Tiedemann study suggest that our findings would likely be similar in other EDs. While patients were prospectively enrolled, patients with less acute conditions were likely consented more often, causing healthier older adults to likely be over-represented and making patient enrollment not truly consecutive. Patients who declined to be in the study were not tracked, making it difficult to get a sense for any self-selection bias. This study relies on older adults’ self-reported fall. Recall bias has been reported in the past when measuring older adults’ reporting of falls.^22^

## CONCLUSION

Future falls and fall-related injuries are high in older adults presenting to the ED. Handgrip strength was not a sensitive screening tool for predicting future falls in older adults. In a validation of Tiedemann et al’s fall-risk screening tool, we found the two-item screening tool was useful to distinguish those at high risk of six- month fall from those at a low risk of falling. EDs may consider using the two-item screening tool developed by Tiedemann et al to assess older adults for future fall risk as it is externally validated and feasible to perform in the ED.

## Supplementary Information



## Figures and Tables

**Table 1:** Overview of Tiedemann score calculation for identifying older adults at risk of future falls.

Tiedemann score	Survey question	Score
Components	“Have you had 2 or more falls in the past 12 months?”	If yes, +2 points
	“Are you taking 6 or more medications?”	If yes, +1 point
Total score	Sum of two scores	Score = 0 to 3

**Table 2:** Subject demographics.

	Total population (N=173)	Fall (n=53)	No fall (n=120)	P-value
Age, median (IQR)	73.5 (69.0 – 80.1)	74.5 (69.5 – 81.0)	73.3 (68.7 – 80.1)	0.297
Gender				0.552
Male, n (%)	79 (45.7)	26 (49.1)	53 (44.2)	
Female, n (%)	94 (54.3)	27 (50.9)	67 (55.8)	
Handgrip test (kg)				
Total				
Dominant grip strength, mean (95% CI)	22.6 (21.0 – 24.2)	20.9 (18.7 – 23.1)	23.4 (21.3 – 25.5)	0.337
Non-dominant grip strength, mean (95%CI)	20.7 (19.1 – 22.3)	20.2 (17.3 – 23.1)	20.9 (19.1 – 22.8)	0.613
Male				
Dominant grip strength, mean (95% CI)	28.5 (25.9 – 31.1)	25.6 (22.6 – 29.0)	29.8 (26.3 – 33.4)	0.103
Non-dominant grip strength, mean (95% CI)	26.4 (24.0 – 28.9)	25.0 (20.4 – 29.5)	27.2 (24.2 – 30.1)	0.117
Female				
Dominant grip strength, mean (95% CI)	17.8 (16.4 – 19.2)	16.6 (14.4 – 18.7)	18.3 (16.5 – 20.1)	0.247
Non-dominant grip strength, mean (95% CI)	16.0 (14.5 – 17.5)	15.5 (12.7 – 18.4)	16.2 (14.4 – 18.0)	0.643
Fall-related current ED visit, n (%)	16 (9.3)	6 (5.0)	10 (18.9)	0.004

*IQR*, interquartile range; *kg,* kilogram; *CI*, confidence interval, *ED*, emergency department.

**Table 3:** Questionnaire results by monthly fall status.

		Falls					

		One month falls		Three month falls		Six month falls	

Question	Yes	n (ROW%)	dOR (95% CI)	n (ROW%)	dOR (95% CI)	n (%)	dOR (95% CI)
Two or more falls in past year	23	9 (39.1)	10.38 (3.34 – 32.23)	7 (30.4)	7.06 (2.19 – 22.78)	16 (69.6)	6.29 (2.37 – 16.68)
Six or more medications	96	10 (10.4)	0.79 (0.27 – 2.34)	8 (8.3)	0.62 (0.20 – 1.91)	39 (40.6)	2.89 (1.26 – 6.64)
Low hand grip strength	54	11 (20.4)	4.30 (1.40 – 13.16)	9 (16.7)	3.36 (1.06 – 10.63)	25 (46.3)	2.47 (1.21 5.06)

*dOR*, diagnostic odds ratio; *CI*, confidence interval.

**Table 4:** Test characteristics to predict 6-month fall outcomes.

	Fall rate, n (%) (48/143, 33.6%)	Sensitivity	Specificity	+LR	−LR	dOR	Injury Rate, n (%) (26/143, 18.2%)	Fall and/or death rate, n (%) (53/173, 30.6%)
Low hand grip strength*	25/54 (46.3%)	52.1% (38.0 – 66.2)	69.5% (60.2 – 78.7)	1.71 (1.01 – 2.41)	0.69 (0.47 – 0.92)	2.47 (1.21 – 5.06)	15/54 (27.8%)	28/70 (40.0%)
Tiedemann’s Screen Score								
3	13/18 (72.2%)	27.1% (14.5 – 39.7)	94.7% (90.3 – 99.2)	5.15 (0.11 – 10.19)	0.77 (0.63 – 0.91)	6.69 (2.22 – 20.14)	7/18 (38.9%)	15/25 (60.0%)
2	3/5 (60.0%)	6.3% (0.0 – 13.1)	97.9% (95.0 – 100.0)	2.97 (−2.29 – 8.22)	0.96 (0.88 – 1.03)	3.10 (0.50 – 3.91)	2/5 (40.0%)	3/10 (30.0%)
≥1	42/101 (41.6%)	87.5% (78.1 – 96.9)	37.9% (28.1 – 47.7)	1.41 (1.14 – 1.68)	0.33 (0.07 – 0.59)	4.27 (1.65 – 11.05)	23/101 (22.8%)	46/128 (35.9%)
1	26/78 (33.3%)	54.2% (40.0 – 68.3)	45.3% (35.3 – 55.3)	0.99 (0.67 – 1.31)	1.01 (0.63 – 1.40)	0.98 (0.49 – 1.96)	14/78 (17.9%)	28/93 (30.1%)
0	6/42(14.3%)	12.5% (3.1 – 21.9)	62.1% (52.4 – 71.9)	0.33 (0.07 – 0.59)	1.41 (1.14 – 1.68)	0.23 (0.09 – 0.61)	3/42 (11.5%)	7/45 (15.6%)

*+LR*, positive likelihood ratio; −*LR*, negative likelihood ratio; *dOR*, diagnostic odds ratio.

* Low handgrip strength defined as a dominant handgrip strength of less than 18 kg (women) and 25 kg (men).
