# Supplementary material for: Two-Item Fall Screening Tool Identifies Older Adults at Increased Risk of Falling after Emergency Department Visit
Source: West J Emerg Med. 2020 Aug 20;21(5):1275–82. doi: 10.5811/westjem.2020.5.46991 (PMC7514384; doi:10.5811/westjem.2020.5.46991)
Supplement: Supplementary file 1 [file wjem-21-1275-s001.docx]

**Supplemental Table S1.** Demographics of Participants by Loss-to-Follow-Up Status.

|  | Analysis Population*  (n=173) | LTFU  (n=73) | Mean Difference  (95%CI) |
| --- | --- | --- | --- |
| **Age**, median (IQR) | 73.5 (69.0 – 80.1) | 73.6 (69.3 – 78.0) | 0.38  (-1.52 to 2.27) |
| **Sex** |  |  | 2.2%  (-11.5 to 16.0) |
| Male, n (%) | 79 (45.7) | 35 (48.0) |  |
| Female, n (%) | 94 (54.3) | 38 (52.1) |  |
| **Hand Grip Test** (kg) |  |  |  |
| *Total* |  |  |  |
| Dominant Grip Strength, mean (95%CI) | 20.6 (16.2 – 26.5) | 18.4 (13.4 – 24.5) | 2.51  (-0.37 to 5.39) |
| Non-Dominant Grip Strength, mean (95%CI) | 19.6 (13.0 – 25.2) | 18.7 (13.0 – 24.6) | 1.56  (-1.19 to 4.31) |
| *Male* |  |  |  |
| Dominant Grip Strength, mean (95%CI) | 26.7 (21.0 – 35.0) | 22.6 (18.2 – 28.3) | 3.70  (-0.86 to 8.25) |
| Non-Dominant Grip Strength, mean (95%CI) | 25.3 (21.6 – 32.1) | 22.8 (17.2 – 28.5) | 3.64  (-0.58 to 7.86) |
| *Female* |  |  |  |
| Dominant Grip Strength, mean (95%CI) | 17.7 (14.1 – 21.7) | 15.5 (10.3 – 19.3) | 1.94  (-0.66 to 4.54) |
| Non-Dominant Grip Strength, mean (95%CI) | 15.5 (11.6 – 20.0) | 15.7 (11.3 – 20.2) | 0.32  (-2.36 to 3.00) |
| **Fall-related Current ED Visit**, n (%) | 16 (9.3) | 12 (16.4) | -7.2%  (-15.9 to 1.5) |

*Includes deaths (n=30)

**Supplemental Table 2.** Questionnaire Results by Monthly Fall Status.

| **Question** | **Yes** | **Falls** | | | | | |
| --- | --- | --- | --- | --- | --- | --- | --- |
|  |  | *One Month Falls* | | *Three Month Falls* | | *Six Month Falls* | |
|  |  | n (ROW%) | dOR (95%CI) | n (ROW%) | dOR (95%CI) | n (ROW%) | dOR (95%CI) |
| Non-Healing Foot Sore | 3 | 0 (0.0) | - | 0 (0.0) | - | 1 (33.3) | 0.99 (0.09 – 11.19) |
| Depressed | 18 | 3 (16.7) | 1.72 (0.44 – 6.75) | 3 (16.7) | 2.07 (0.52 – 8.28) | 9 (50.0) | 2.21 (0.81 – 5.98) |
| Unable to Clip Own Toenails | 38 | 7 (18.4) | 2.41 (0.83 – 7.00) | 6 (15.8) | 2.27 (0.73 – 7.05) | 21 (55.3) | 3.57 (1.64 – 7.75) |
| Two or More Falls in Past Yr. | 23 | 9 (39.1) | 10.38 (3.34 – 32.23) | 7 (30.4) | 7.06 (2.19 – 22.78) | 16 (69.6) | 6.29 (2.37 – 16.68) |
| Six or More Medications | 96 | 10 (10.4) | 0.79 (0.27 – 2.34) | 8 (8.3) | 0.62 (0.20 – 1.91) | 39 (40.6) | 2.89 (1.26 – 6.64) |
| Low Hand Grip Strength | 54 | 11 (20.4) | 4.30 (1.40 – 13.16) | 9 (16.7) | 3.36 (1.06 – 10.63) | 25 (46.3) | 2.47 (1.21 5.06) |

**Supplemental Table 3.** Test Characteristics to predict 6-month fall outcomes.

| *Sensitivity Analysis of Falls and Deaths with LTFU population** | | | | | |
| --- | --- | --- | --- | --- | --- |
|  | **Sensitivity** | **Specificity** | **+LR** | **-LR** | **dOR** |
| Handgrip Strength | 52.6%  (41.5 – 63.7) | 57.7%  (50.3 – 65.2) | 1.24  (0.90 – 1.59) | 0.82  (0.60 – 1.04) | 1.51  (0.88 – 2.60) |
| Tiedemann’s Screen (Score of 3) | 25.6%  (16.0 – 35.3) | 88.1%  (83.2 – 93.0) | 2.15  (0.95 – 3.36) | 0.84  (0.72 – 0.96) | 2.55  (1.28 – 5.09) |
| Two Falls in Past Year (Score of 2) | 35.9%  (25.3 - 46.5) | 83.9%  (78.4 – 89.5) | 2.23  (1.21 – 3.26) | 0.76  (0.63 – 0.90) | 2.92  (1.57 – 5.43) |
| Six or More Meds (Score of 1) | 78.2%  (69.0 – 87.4) | 34.5%  (27.3 – 41.7) | 1.19  (1.00 – 1.39) | 0.63  (0.33 – 0.93) | 1.89  (1.01 – 3.55) |
| Negative Tiedemann’s Screen (Score of 0) | 11.5%  (4.5 – 18.6) | 69.6%  (62.7 – 76.6) | 0.38  (0.13 – 0.63) | 1.27  (1.11 – 1.43) | 0.30  (0.14 – 0.65) |

**n=246, all lost to follow-up participants assumed as no fall.*
